# Mental Ill‐Health in Mothers Caring for Offspring With Intellectual Disabilities at Different Stages of Caregiving: Secondary Data Analysis and Data Linkage of Administrative and Health Records

**DOI:** 10.1111/jar.70200

**Published:** 2026-02-18

**Authors:** Deborah Cairns, Kirsty Dunn, Robin Young, Nicola Greenlaw, Sally Ann Cooper, Jill P. Pell, Ewelina Rydzewska

**Affiliations:** ^1^ University of Glasgow Glasgow Scotland UK; ^2^ University of Edinburgh Edinburgh Scotland UK

**Keywords:** caregiving, carer, death, intellectual disability, mental ill‐health, mother, offspring

## Abstract

**Background:**

Little research has investigated maternal‐carer mental ill‐health at different stages of care‐giving, including following the death of offspring with intellectual disabilities.

**Methods:**

Population cohort study of 9787 mothers of offspring with intellectual disabilities, matched with 30,235 mothers of offspring without intellectual disabilities.

**Results:**

Mental ill‐health was significantly higher for mothers of offspring with intellectual disabilities (OR = 1.28, 95% CI [1.22–1.34]) than mothers of offspring without intellectual disabilities and at different stages: child (OR = 1.40, 95% CI [1.30–1.51]), adult (OR = 1.22 95% CI [1.14–1.29]) but not older adults (OR = 1.22, 95% CI [0.91–1.65]). Mothers with a mental health diagnosis, compared to those without, were significantly more likely to have long‐term health problems, poorer health and socioeconomic circumstances (e.g., greater neighbourhood deprivation) (all at *p* < 0.0001). No difference was found between mothers' mental health whose offspring with/without intellectual disabilities died (*p* = 0.68).

**Conclusions:**

This study provides unique insights into factors associated with the mental health of maternal carers and the need for services to better meet their needs.

## Lay Summary

- Mothers of offspring with intellectual disabilities were more likely to experience mental ill‐health compared to mothers of offspring without intellectual disabilities.
- Among mothers of offspring with intellectual disabilities, those who had mental ill‐health were more likely to experience long term health problems and experience poorer socioeconomic circumstances (e.g., greater neighbourhood deprivation) compared to those who did not have mental ill‐health.
- Mental ill‐health was similar for mothers whose offspring with (40.5%) or without intellectual disabilities (38.3%) had died.
- Support for maternal carers is needed throughout the caregiving journey, given the high rates of mental ill‐health.

## 1 Background

Improvements in health care around the world and deinstitutionalisation has led to more people with intellectual disabilities living in the community and experiencing longer, happier lives (McCarron et al. 2019). This has meant that parents are increasingly providing support for their children for longer periods. While this can be an overall positive and very rewarding experience (Jokinen and Brown 2005), studies suggest it may also have a negative impact on parents' mental health at different points in caregiving (caring for a child, adult, older adult) (Emerson and Hatton 2011), including after the death of the care recipient (described hereafter as ‘post caregiving’) (Totsika, Hastings, Emerson, Lancaster, et al. 2011a; Totsika, Hastings, Emerson, Berridge, et al. 2011b).

Scotland's Census data from 2011 reports that there are 26,349 (0.5%) people with intellectual disabilities (5234 children (0.6%) and 21,115 adults (0.5%)) who reside in Scotland (National Records of Scotland 2011). Four thousand and fifty‐one children (77.4%) and an anticipated 7000 adults (35%) with intellectual disabilities live with a parent carer, who plays a substantial role in supporting them (National Records of Scotland 2011).

There is some evidence to suggest that mothers caring for a son or daughter (offspring) with intellectual disabilities report poorer mental health compared to fathers (Dunn et al. 2019) and other carer groups (e.g., a son/daughter providing support to an elderly parent with dementia or a parent supporting a child with physical disabilities) (Pinquart and Sörensen 2003) and may be more susceptible to depression or anxiety (Gogoi et al. 2017). These studies address an important and under‐researched area and one that requires further investigation. A review of the literature identified that studies focused only on specific factors such as the mental health of maternal carers of children with specific types (e.g., Down syndrome) or levels of disability (Nitta et al. 2007; Emerson 2003). Other studies focused only on the mental health of parent carers within certain age groups (e.g., older parent carers) (Totsika, Hastings, Emerson, Berridge, et al. 2011b; Cairns et al. 2014; Heifetz et al. 2019) and some quantitative studies used small samples (Cairns et al. 2014; Bourke‐Taylor et al. 2012; Bourke et al. 2008); there are subsequently gaps in the literature. A recent systematic review and meta‐analyses of evidence on the mental health of mothers caring for offspring with intellectual disabilities found that they experienced poorer mental health compared to mothers of typically developing people. Meta‐analyses revealed significant findings for anxiety, depression, parenting stress, emotional burden and common mental disorders (Rydzweska et al. 2021). The review also reinforced that the quality of existing research is limited, particularly regarding small sample sizes, biased sample recruitment, inclusion criteria, and that there is scarce research on mental ill‐health of mothers of adults with intellectual disabilities across the caregiving trajectory.

According to the Mental Health Foundation (2015), who take a ‘life course’ approach to mental health, each stage of the life course (e.g., the early years, adulthood and later life) presents different challenges as well as opportunities to intervene and support good mental health. Mental health problems are one of the main causes of the burden of disease worldwide (Vos et al. 2013) and one in four people in the UK will experience a mental health problem in any given year (The Scottish Government 2025; NHS England 2026). A systematic review of 31 studies estimated that in the UK, more mothers (10%) than fathers (6%) had mental health problems at any given time (Parker et al. 2008). The duration of caregiving may also compound or mitigate against mental ill‐health. Being a parent carer of a child with intellectual disabilities over a prolonged period is a very different experience to taking on a caring role, for example, for someone who has become seriously ill (Pattison et al. 2021). Prolonged caregiving may be a particular risk for mental ill‐health (Cairns et al. 2014; Pattison et al. 2021). However, there is a clear lack of robust evidence on the prevalence and determinants of mental ill‐health in maternal carers of offspring with intellectual disabilities across the different stages of caregiving (early years (child), adulthood, and later life), including post caregiving.

Life expectancy of people with intellectual disabilities is lower than the rest of the population (O'Leary et al. 2018) so mothers can and do outlive their child, and this might affect their mental health. A recent cohort study, with record linkage to death data, investigating mortality in adults with intellectual disabilities (*n* = 962) in Scotland, reported that adults with intellectual disabilities are twice as likely (SMR = 2.24) to die from preventable illnesses compared to their peers without intellectual disabilities (Cooper et al. 2020). Hosking et al. (2016) reported that the crude mortality rate was 132.4/10,000 people per year among adults with intellectual disabilities, compared with 39.7/10,000 among the UK general population. A more recent retrospective cohort study, comparing Scottish Census 2011 data for 7247 children with intellectual disabilities (aged 5–24 years) with that of 156,439 children without intellectual disabilities, and linking to the death register, reported that children with intellectual disabilities in Scotland were at least 11 times more likely to die than their peers without intellectual disabilities from illnesses that could have been prevented or treated (Hughes‐McCormack et al. 2022). While bereavement is difficult for anyone, those providing unpaid care to a family member may be affected differently (Hawton 2007). Several studies have focused on carer bereavement of family members with, e.g., dementia (Boerner and Schulz 2009) or cancer (Chiu et al. 2010), but there is a research gap on the mental health of mothers after the death of an offspring with intellectual disabilities.

In summary, there is a lack of robust research investigating mental ill‐health prevalence in maternal carers of offspring with intellectual disabilities at different stages of caregiving, including post caregiving, and the factors that compound or mitigate against it. The dearth of empirical evidence that explores the intersections of gender, class and disability presents a barrier to understanding the complex factors that produce differential individual health outcomes. The aim of this paper was to investigate the prevalence and factors associated with mental ill‐health in mothers caring for offspring with intellectual disabilities, both overall and at different stages of caregiving, including post caregiving.

## 2 Research Questions

1. Are there associations between mothers caring for offspring with intellectual disabilities and the presence of mental ill‐health at different stages of caregiving?
2. Is there an association between the death of an offspring with intellectual disabilities and the presence of mental ill‐health in mother caregivers?
3. To what extent are individual (e.g., social class, education, marital status), household (e.g., type of accommodation, rurality) and offspring (e.g., gender, mental ill‐health) characteristics associated with the risks of mental ill‐health in mothers' overall and at different stages of caregiving, including post caregiving?

*Approvals*: National Health Service (NHS) Ethics, Administrative Data Research Network (ADRN), Public Benefit, Privacy Panel for Health and Social Care and University of Glasgow Ethical approvals were obtained in 2017 and again in 2022 (post‐COVID 19, due to the delay in access to record‐linked data as COVID 19 research was nationally prioritised above other projects).

## 3 Methods

Scotland's Census 2011 was used to ascertain all mothers caring for offspring with intellectual disabilities. Scotland's Census is unique worldwide in including a question on ‘intellectual disability’ and specifically separating this from specific learning difficulties (e.g., dyslexia and dyscalculia), and from autism. There is also a question asking individuals if they have a mental health condition that has lasted, or is expected to last, at least 12 months. Cognitive question testing with retrospective probing was undertaken prior to the Census, to test whether the self/proxy question on ‘mental health condition’ was answered accurately and willingly by respondents, and to identify what changes might be required to improve data quality and/or the acceptability of the response options (see Cooper et al. 2022 for full details). For the first time, mothers with a caring role can be identified, as can households with a child or adult with intellectual disabilities, and the individual characteristics of both mother and child can be linked. It hence provides a unique and valuable resource to identify the mental health of maternal carers of offspring with intellectual disabilities. Scotland's Census 2011 is the most recent available data that is accessible. This population cohort study analysed linked routinely collected data, from different sources. Mothers' mental ill‐health was determined via: self‐report (Scotland's Census); use of hypnotic, anxiolytic and antidepressant medication (Prescribing Information System); and psychiatric hospital admissions (Scottish Morbidity Records).

Mothers caring for offspring with intellectual disabilities were matched 3:1 with mothers of offspring who did not have intellectual disabilities by mother's age, neighbourhood deprivation (SIMD) and gender of child (depending on research question). The extent to which individual, household and child characteristics mitigated or compounded the risk of mental ill‐health in mothers overall and at different stages of caregiving, including post caregiving, were also investigated. The stages of caregiving were categorised into three groups, adapted from Emerson and Hatton (2011):

1. Caring for a child (0–15 years),
2. Caring for an adult (16–54 years),
3. Caring for an older adult (55+ years).

‘Older adults’ are categorised as 55+ years of age as it is generally accepted that individuals with intellectual disabilities, not just people with Down syndrome, show signs of premature ageing (Perkins and Moran 2010).

The outcome was ascertained from three sources (self‐report, medications, hospital admissions), in which the mother is considered as having a mental health condition if at least one of the sources confirms so. For outcomes following the death of the child post Census 2011, the mental health of the mothers was assessed on medication use and psychiatric hospital admissions only.

### 3.1 Data Sources

*Scotland's Census 2011* identified mothers caring for offspring with intellectual disabilities living at home; mothers of offspring without intellectual disabilities living at home; mental health condition status; and individual, household, and child characteristics (e.g., mental health condition status). It is the only survey which provides a detailed picture of the entire population. It is unique nationally as it covers everyone at the same time (27th March 2011) and asks the same core questions of everyone (e.g., age, gender, marital status, employment status). It is unique internationally, as it asks about intellectual disabilities (and distinguishes this from specific learning difficulties such as dyslexia) and also includes information about long‐term conditions including if an individual identifies as having a mental health condition, or developmental disorder (e.g., autistic spectrum disorder or Asperger's syndrome), is blind or sight loss, deaf or hearing impairments, and a general health rating. Scotland's Census 2011 was completed by 94% of the whole of Scotland's population.

*Community Health Index (CHI) Database* was used to CHI seed the study individuals included from the Census 2011. The CHI number is a unique identifier for each person in Scotland and is included in all health databases, enabling linkage between these databases and covers 97% of Scotland's population.

*Prescribing Information System (PIS)* identified mothers' mental ill‐health from use of hypnotic, anxiolytic and antidepressant medication from 27th March 2010 (1 year prior to Census completion) up to the end of 2017, to compare mental ill‐health pre and post bereavement. PIS includes names of all drugs from dispensed prescriptions. The prescriptions include the patients' unique CHI number; hence prescriptions can be linked at the individual level with the other datasets using the CHI number.

*Scottish Morbidity Records 04 (SMR04)—Mental Health Inpatient and Day Case* identified mothers who received psychiatric inpatient or day care, from 27th March 2010 (1 year prior to Census completion) up to the end of 2017. SMR04 includes episode level data, and patients CHI number, hence enabling linkage. It also includes a wide variety of information, e.g., patient characteristics and diagnosis at episode level.

*National Records Scotland (NRS) Register of deaths* was used to identify deaths of people with intellectual disabilities between Scotland's Census 2011 (28th March 2011) and up to the end of 2017 using their CHI number, allowing mothers' mental ill‐health to be compared pre and post bereavement using PIS and SMR04 data. The registration of a death in Scotland is controlled by the Registration of Births, Deaths and Marriages (Scotland) Act 1965. The register includes CHI number.

#### 3.1.1 Record Linkage

Variables from CHI were used for processing only: name, month and year of birth, postcode, to CHI seed participants from the Census. CHI number was linked to Census variables to PIS, SMR04, and NRS death data (see Supporting Information for list of variables and codes). None of these variables, nor CHI, were released to the research team.

#### 3.1.2 Data Analysis

Data was pseudonymised prior to being made available to the research team so individuals could not be identified by those undertaking any analysis. Incidence for mental health condition/s was calculated with 95% confidence intervals for mothers of offspring with intellectual disabilities and mothers with offspring without intellectual disabilities, overall, and at each stage of caregiving including post caregiving. Characteristics of the exposed (mothers caring for offspring with intellectual disabilities) and unexposed (mothers caring for offspring without intellectual disabilities) groups were presented within descriptive tables. Comparisons between groups were made using chi‐square tests for categorical data, and *t*‐tests for continuous data. Logistic regression was used to calculate the odds of mothers experiencing mental ill‐health. Software R version 3 was used to conduct the analysis (R Core Team 2021).

## 4 Results

**FIGURE 1:**
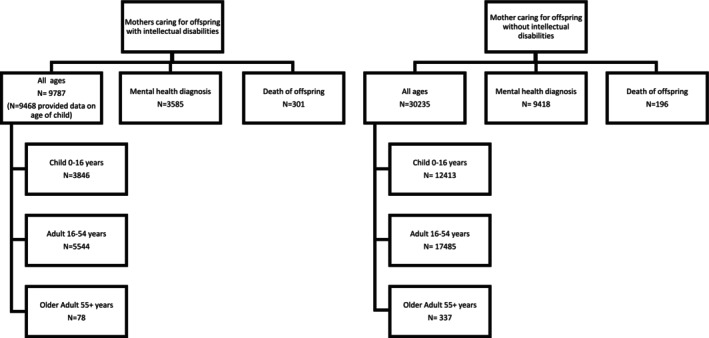
Flowchart of subgroups in the sample.

Scotland's Census identified a total of 9787 mothers caring for offspring (child, adult, older adult) with intellectual disabilities and 30,235 mothers caring for offspring without intellectual disabilities (see Table 1 and Figure 1 for characteristics of sample).

Research Question 1 *Associations between mothers caring for offspring with intellectual disabilities and the presence of mental ill‐health at different stages of caregiving*.

**TABLE 1:** Characteristics of the sample.

	Mothers caring for offspring with intellectual disabilities, *N* = 9787	Mothers caring for offspring without intellectual disabilities, *N* = 30,235
Offspring characteristics		
Offspring mean age (SD)	20.8 (13.2)	20.1 (13.4)
Offspring sex (male)	5636 (59.9%)	18,034 (59.6%)
Offspring with mental ill‐health	2739 (28.9%)	4147 (13.7%)
Offspring died	301 (3.1%)	196 (0.6%)
Mother characteristics		
Mother mean age (SD)	49.6 (13.2)	49.5 (13.2)
Employed	5803 (59.3%)	25,297 (83.7%)
Provision of unpaid care		
1–49 h per week	1340 (13.7%)	3433 (11.4%)
50+ hours per week	6977 (71.3%)	1416 (4.7%)

A mental ill‐health record existed for 3585 (36.6%) mothers of with intellectual disabilities, compared to 9418 (31.1%) mothers of offspring without intellectual disabilities. Overall, there was a significant difference in the odds of experiencing mental ill‐health between mothers who did and did not have offspring with intellectual disabilities, with the odds being higher for the mothers of offspring with intellectual disabilities (OR = 1.28, 95% CI [1.22–1.34], *p* < 0.0001). When comparing mothers at different stages of caregiving, data on child age was available for 9468 mothers. Differences between groups were detected at various stages; child 0–15 years (OR = 1.4035, 95% CI [1.3045–1.5101], *p* < 0.0001) and adult 16–54 years (OR = 1.22, 95% CI [1.14–1.29], *p* < 0.0001). However, there was no significant difference in the odds of experiencing mental ill‐health between mothers who did and did not have offspring with intellectual disabilities for older adults 55+ years (OR = 1.22, 95% CI [0.91–1.65], *p* = 0.19) (see Table 2).

Research Question 2 *Associations between the death of an offspring with intellectual disabilities and the presence of mental ill‐health in mother caregivers*.

**TABLE 2:** Odds of experiencing mental ill‐health for mothers with or without offspring with intellectual disabilities by caregiving stage.

Caregiving stage	*N*	Odds ratio	95% CI	*p*
All ages	40,022	1.2777	[1.218–1.3402]	< 0.0001
0–15	16,259	1.4035	[1.3045–1.5101]	< 0.0001
16–54	23,029	1.2155	[1.1422–1.2936]	< 0.0001
55+	415	1.2239	[0.9063–1.6526]	0.1874

*Note:* Univariate logistic regression models were run for all ages combined and also separately within each caregiving stage. The reference category for all of the four separate analysis is ‘Mothers without offspring with Intellectual disability’.

Significantly more mothers of offspring with intellectual disabilities were reported to have offspring who had died (*N* = 301, 3.1%) than mothers of offspring without intellectual disabilities (*N* = 196, 0.6%, *p* < 0.0001). However, there was no statistically significant difference in mental ill‐health between mothers of offspring, who did or did not have intellectual disabilities, who had died. Of the mothers of offspring with intellectual disabilities who died, 122 (40.5%) had mental ill‐health. This applied to 75 (38.3%) mothers of offspring without intellectual disabilities who died (*p* = 0.68) (Table 3).

Research Question 3 *Characteristics associated with the risk of mental ill‐health in mothers' overall and at different stages of caregiving, including post caregiving*.

**TABLE 3:** Death of offspring.

	Mothers caring for offspring with intellectual disabilities, *N* = 9787	Mothers caring for offspring without intellectual disabilities, *N* = 30,235	*p*
Death of offspring	301 (3.1%)	196 (0.6%)	< 0.0001

	Mothers whose offspring with intellectual disabilities died, *N* = 301	Mothers whose offspring without intellectual disabilities died, *N* = 196	*p*
Mental ill‐health	122 (40.5%)	75 (38.3%)	0.6811

### 4.1 Mothers of Offspring With Intellectual Disabilities

Statistically significant differences were found on several variables between mothers of offspring with intellectual disabilities who did and did not have mental ill‐health. Mothers with mental ill‐health were more likely to have offspring with mental ill‐health (33.0% vs. 26.6%, *p* < 0.0001), to have a long‐term health problem (55.5% vs. 33.0%, *p* < 0.0001), and to be from the most deprived socioeconomic area (SIMD 1–2) (28.9% vs. 21.2%, *p* < 0.0001). They were less likely to have a higher level of education (36.7% vs. 42.7%, *p* < 0.0001), to describe their general health as good or very good (59.7% vs. 77.5%, *p* < 0.0001), to describe the general health of their offspring as good or very good (47.3% vs. 54.3%, *p* < 0.0001), and to be married or in a civil partnership (53.6% vs. 62.2%, *p* < 0.0001) (Table 4).

**TABLE 4:** Mothers of offspring with intellectual disabilities with and without mental ill‐health.

	Mothers with mental ill‐health, *N* = 3585	Mothers without mental ill‐health, *N* = 6202	*p*
Child characteristics			
Offspring with mental ill‐health, *N* (%)	1140 (33.0%)	1599 (26.6%)	< 0.0001
General health of offspring described as good or very good, *N* (%)	1632 (47.3%)	3266 (54.3%)	< 0.0001
Mother characteristics			
Long‐term health problem, *N* (%)	1990 (55.5%)	2048 (33.0%)	< 0.0001
General health as very good or good, *N* (%)	2139 (59.7%)	4806 (77.5%)	< 0.0001
Most deprived socioeconomic area (SIMD 1–2), *N* (%)	1036 (28.9%)	1317 (21.2%)	< 0.0001
Higher level of education (Scottish Highers or above), *N* (%)	1314 (36.7%)	2646 (42.7%)	< 0.0001
Married or in a civil partnership, *N* (%)	1920 (53.6%)	3855 (62.2%)	< 0.0001

### 4.2 Mothers of Offspring Without Intellectual Disabilities

Similarly, statistically significant differences were found on several variables between mothers of offspring without intellectual disabilities who did and did not have mental ill‐health. Mothers with mental ill‐health were more likely to have offspring with mental ill‐health (16.9% vs. 12.3%, *p* < 0.0001), to have a long‐term health problem (46.0% vs. 26.2%, *p* < 0.0001), to be from the most deprived socioeconomic area (SIMD 1–2) (28.9% vs. 22.0%, *p* < 0.0001), and to provide unpaid care (17.8% vs. 15.2%, *p* < 0.0001). They were less likely to have a higher level of education (42.2% vs. 49.6%, *p* < 0.0001), to describe their general health as good or very good (68.0% vs. 84.3%, *p* < 0.0001), to describe the general health of their offspring as good or very good (91.8% vs. 94.2%, *p* < 0.0001), and to be married or in a civil partnership (53.5% vs. 64.4%, *p* < 0.0001) (Table 5).

**TABLE 5:** Mothers of offspring without intellectual disabilities with and without mental ill‐health.

	Mothers with mental ill‐health, *N* = 9418	Mothers without mental ill‐health, *N* = 20,817	*p*
Offspring characteristics			
Offspring with mental ill‐health, *N* (%)	1589 (16.9%)	2558 (12.3%)	< 0.0001
General health of offspring described as good or very good, *N* (%)	8643 (91.8%)	19,613 (94.2%)	< 0.0001
Mother characteristics			
Long‐term health problem, *N* (%)	4331 (46.0%)	5438 (26.2%)	< 0.0001
General health as very good or good, *N* (%)	6405 (68.0%)	17,550 (84.3%)	< 0.0001
Most deprived socioeconomic area (SIMD 1–2), *N* (%)	2725 (28.9%)	4583 (22.0%)	< 0.0001
Higher level of education (Scottish Highers or above), *N* (%)	3974 (42.2%)	10,332 (49.6%)	< 0.0001
Provision of unpaid care, *N* (%)	1680 (17.8%)	3169 (15.2%)	< 0.0001
Married or in a civil partnership, *N* (%)	5035 (53.5%)	13,396 (64.4%)	< 0.0001

## 5 Discussion

This is the first known study to investigate the mental health of mothers of offspring with intellectual disabilities at different stages of caregiving, including post caregiving. Doing so provides an insight into factors associated with mental ill‐health at different stages of life. Our study reported a significant difference in the odds of experiencing mental ill‐health between mothers who did and did not have offspring with intellectual disabilities. When comparing mothers at different stages of the caregiving journey, significant differences between groups were also detected in both children (0–15 years) and adults (16–54 years). However, there was no significant difference in the odds of experiencing mental ill‐health between mothers who did and did not have offspring with intellectual disabilities for older adults (55+ years). There was also no statistically significant difference in mental ill‐health diagnosis between mothers whose offspring with/without intellectual disabilities had died. These findings shall now be discussed in turn and discussed within the context of the wider literature.

### 5.1 Caring for Offspring (0–15 Years)

Our study found that mothers of children (0–15 years) with intellectual disabilities were significantly more likely to experience mental ill‐health compared to mothers of children without intellectual disabilities. These findings resonate with a recent systematic review and meta‐analysis (Masefield et al. 2020) which found an association between mothers caring for preschool children with a developmental disability and poorer ill‐health (standardised mean difference 0.87; 95% predictive interval −0.47, 2.22). In a further systematic review investigating depression and anxiety in parents of children (aged < 18) with intellectual and developmental disabilities (Scherer et al. 2019), nearly all studies found a positive association between parenting a child with intellectual and developmental disabilities and depression (*n* = 18, 95%) and anxiety (*n* = 9, 90%) symptoms. Factors associated with higher levels of depression symptoms among parents of children with intellectual and developmental disabilities included disability severity (*n* = 8, 78%) and lower household income (*n* = 4, 80%). Most studies in this systematic review reported higher levels of depression in mothers compared to fathers.

While raising a child with an intellectual disability can be an extremely rewarding and positive experience (Hastings et al. 2002; Beighton and Wills 2017), there are chronic stressors inherent in raising a child with an intellectual disability, including high caregiver demands (Raina et al. 2004) and financial strain (Parish et al. 2008). As a result of these stressors, parents of children with intellectual disabilities may be more susceptible to depression and anxiety (Olsson and Hwang 2001). These experiences are not dissimilar when caring for a child with intellectual disabilities into adolescence.

Adolescence is a critical period of development and transition (Jordan and Andersen 2017; Backes et al. 2019). Adolescents with intellectual disabilities face various challenges and risks, such as educational difficulties, social isolation, stigma and discrimination, which affect their self‐esteem, identity, health and well‐being (Christensen et al. 2012). Mothers of adolescents with intellectual disabilities play a vital role in providing care and support for their offspring, and in facilitating their development and transition (Kerr et al. 2023; Bourke‐Taylor et al. 2021). Mothers of adolescents with intellectual disabilities also experience various stressors and challenges, such as emotional distress, financial burden and social stigma which can have an impact on their mental health and quality of life (Staunton et al. 2023); these challenges often continue as the offspring with intellectual disabilities move into adulthood.

### 5.2 Caring for Adult Offspring (16–54 Years)

The current study found mothers caring for adult offspring with intellectual disabilities (16–54 years) experienced poorer mental health compared to mothers of offspring without intellectual disabilities. People with intellectual disabilities are enjoying longer lives due to improvements in health care, medical technology, nutrition and living standards (Redley 2019). However, they experience greater health and social inequalities that lead to significant and extended caring roles for parents who themselves are ageing (Redley 2019). Older parent‐carers therefore not only experience their own age‐related conditions but also those of their offspring, and vice versa. While carers experience satisfaction with their role, small scale studies suggest stress is also common and exacerbated with age, negatively affecting physical and mental health of older parent‐carers and/or their offspring with intellectual disabilities (Totsika, Hastings, Emerson, Lancaster, et al. 2011a; Totsika, Hastings, Emerson, Berridge, et al. 2011b; Cairns et al. 2014). It is therefore unsurprising that mothers of adult children with intellectual disabilities are thought to be at risk of poorer health in later life (Totsika, Hastings, Emerson, Lancaster, et al. 2011a; Totsika, Hastings, Emerson, Berridge, et al. 2011b; Cairns et al. 2014).

### 5.3 Caring for Older Adult Offspring (55 + Years)

Our study found no significant difference in the odds of experiencing mental ill‐health between mothers who did and did not have offspring with intellectual disabilities for older adults (55+ years) and there were fewer mother carers looking after a child into old age. This is possibly due to the lower sample size. However, it may also be that the older adults with intellectual disabilities who have survived, are most likely to be individuals with mild intellectual disabilities, with less complex needs (Cooper et al. 2015) and who therefore require less support from their ageing parents. An exploratory survey on the experiences and health of older parent carers living in Scotland (Cairns et al. 2014) found that older parent carers (65 years and older) had above average mental health compared to the general population. A possible factor is that older parents' caregiving experiences may be enhanced by increasing reciprocity with their ageing offspring with intellectual disabilities taking on some caring roles for their parents, which may in turn result in health benefits (Gant 2010).

### 5.4 Death of Offspring

Our study found that the death of an offspring was significantly higher in more mothers of offspring with intellectual disabilities (*N* = 301, 3.1%) compared to mothers of offspring without intellectual disabilities (*N* = 196, 0.6%). People with intellectual disabilities have a significantly higher prevalence of physical and mental ill‐health (Kinnear et al. 2018; Cooper et al. 2015), dying on between 15 and 20 years younger compared to the general population (O'Leary et al. 2018; Rydzewska et al. 2025); it is therefore not surprising that more offspring with intellectual disabilities died.

There was no statistically significant difference in mental ill‐health diagnosis between offspring with/without intellectual disabilities whose offspring had died. Of the mothers of offspring with intellectual disabilities who died, 122 (40.5%) had a diagnosis of mental ill‐health and 75 (38.3%) mothers of offspring without intellectual disabilities who died had a diagnosis of mental ill‐health. The death of an offspring to any mother, regardless of disability, is undoubtedly going to be a devastating experience for a parent. The grief experienced by parents is more intense and prolonged in comparison to grief experienced following the death of other family members (Middleton et al. 1998), believed to be due to the close relationships parents have with their children (Kreicbergs et al. 2004). Parents are not expected to outlive their children and when this happens, it can have a significant impact on the parents' mental health; research of bereaved parents in general reports a higher risk for complicated grief (Meert et al. 2010), anxiety and depression (Kreicbergs et al. 2004), and post‐traumatic stress disorder (Murphy et al. 2003).

### 5.5 Characteristics Associated With Mother's Mental Health

Significant differences were also found on several variables between mothers of offspring with intellectual disabilities who did and did not have a mental health diagnosis. Mothers with a mental health diagnosis were more likely to have offspring with a mental health diagnosis, to have a long‐term health problem, and to be from the most deprived socioeconomic area. They were less likely to have a higher level of education, to describe their general health as very good or good, to describe the general health of their offspring as good or very good, and to be married or in a civil partnership.

These findings reinforce that social, psychological, biological and environmental factors interact with and impact on an individual's mental health, with the poorer and more disadvantaged disproportionately affected by common mental health problems. As previously reported, these studies suggest that factors such as the mental health of children, marital status, socio‐economic status and parent educational levels may play a role in mental ill‐health in general. For mothers of offspring with intellectual disabilities, studies have reported a relationship between marital status and stress or depression, with single caregivers experiencing more stress than caregivers in a married or common–law relationship (Marquis et al. 2019). Eisenhower and Blacher (2006) found that being currently employed and being married predicted significantly better psychological well‐being for parents of offspring with intellectual and developmental disabilities. Eisenhower et al. (2009) also reported that higher maternal education was associated with better self‐reported health in mothers of offspring with intellectual and developmental disabilities. Socio‐economic hardship has also been associated with negative outcomes for female carers' well‐being (Chou et al. 2010). Emerson (2003) found that families supporting offspring with intellectual disabilities were significantly more economically disadvantaged compared to families supporting offspring without intellectual disabilities; mothers' mental health problems were also associated with various factors such as poverty and receipt of means‐tested welfare benefits.

Parents caring for offspring with intellectual disabilities also have poorer physical health with higher incidence of, for example, sleep disturbance, gastrointestinal problems and high blood pressure (Gallagher and Whiteley 2012, 2013). It is therefore possible that the increased risk in poor mental health seen in mothers with offspring with intellectual disabilities may be partly explained by their physical health in addition to the aforementioned factors (e.g., offspring, marital status, finances).

## 6 Strengths and Limitations

Scotland's Census investigated the whole population (5.3 million), which is one of the biggest population sizes to date. Scotland's Census is unique as it systematically enquired about each person living at home with the condition intellectual disabilities. In addition, the phrasing of the questions underwent cognitive question testing prior to the Census, ensuring the intended meaning was captured. 94% of the population completed the Census, and the record linkage was successful in a high percentage. The authors believe these results are therefore generalisable to other high‐income countries, in addition to filling a significant gap in existing literature on the mental health of mothers of children with intellectual disabilities across and beyond caregiving (for further details on Scotland's Census: Cooper et al. 2022).

Based on the data available, we do not know the time scale of when mothers experienced mental ill‐health in relation to the death of their offspring, or in relation to the age of their offspring dying and if mental ill‐health is actually associated with the death of the offspring or that of additional factors, although the research evidence, as previously discussed, does suggest that mental ill‐health is exacerbated in mothers following the death of an offspring in any circumstances. We must also acknowledge that not all mental health diagnoses are medicated, and not all individuals will be referred as in‐patients. Furthermore, some psychotropic medications are prescribed for non‐mental health reasons, such as chronic pain, which could subsequently lead to misclassification of some participants.

## 7 Implications

This study provides unique insights into the multiple social determinants of health that impact on mental health outcomes of maternal carers and contribute to building the empirical evidence on intersectionality. This is needed so services can deliver the right support, in the right place, when it is most needed. This pioneering research is set in Scotland's rich informatics environment, linking existing routinely collected large datasets to benefit mothers caring for offspring with intellectual disabilities and mothers post caregiving, across all social classes. It shows that support for maternal carers is needed well into, and throughout, their offspring's adulthood and middle age, given their higher rates of mental ill‐health.

These findings also enrich our understanding of the complex interrelationship between socio‐economic factors and caregiving. We have addressed important and overdue questions on mental ill‐health to inform professional support for mothers at pivotal points in the caregiving journey including post caregiving. Without adequate formal support, carers' mental health may be at risk and without information on the prevalence and determinants of mental ill‐health in maternal carers, formal support cannot be tailored to meet their needs in an appropriate and timely manner.

The proposed research raises national and international awareness of the mental health of maternal carers of offspring with intellectual disabilities at different stages of the caregiving journey and post caregiving. These findings can inform professional understanding of when additional support is required to prevent/ameliorate mental ill‐health and inform policymakers and service commissioners what formal supports mothers need in order to address/prevent mental ill‐health.

It is hoped these findings will provide impetus for change in public spending, from a focus almost entirely on coping with crisis (Balmer 2015), to an investment in preventive or anticipatory strategies. We also hope that the uniqueness and benefits of Scotland's Census 2011 question on intellectual disabilities will inspire other countries to include the same question in future Censuses and that the methods used in this study will enthuse researchers to investigate an area that is of international importance. This research is the first to use Scotland's routinely collected health data to bring benefits for maternal carers of offspring with intellectual disabilities.

## Author Contributions

Deborah Cairns conceived the study. Deborah Cairns, Sally Ann Cooper, Jill P. Pell, Nicola Greenlaw and Ewelina Rydzewska were involved in the design of the study. Robin Young led the analysis of the data. Deborah Cairns and Kirsty Dunn prepared the first draft of the manuscript. All authors contributed to the manuscript writing. All authors approved the final manuscript. Deborah Cairns is the study guarantor.

## Funding

This research was funded by ESRC Secondary Data Analysis Initiative.

## Ethics Statement

National Health Service (NHS) Ethics, Administrative Data Research Network (ADRN), Public Benefit, Privacy Panel for Health and Social Care and University of Glasgow Ethical approvals were obtained in 2017 and again in 2022 (post‐COVID 19, due to the delay in access to record‐linked data as COVID 19 research was nationally prioritised above other projects).

## Conflicts of Interest

The authors declare no conflicts of interest.

## Supporting information


**Data S1:** jar70200‐sup‐0001‐Suppinfo.docx.
