# Supplementary material for: Mental Ill‐Health in Mothers Caring for Offspring With Intellectual Disabilities at Different Stages of Caregiving: Secondary Data Analysis and Data Linkage of Administrative and Health Records
Source: J Appl Res Intellect Disabil. 2026 Feb 18;39(1):e70200. doi: 10.1111/jar.70200 (PMC12916229; doi:10.1111/jar.70200)
Supplement: Supplementary file 1 — Data S1: jar70200‐sup‐0001‐Suppinfo.docx. [file JAR-39-e70200-s001.docx]

**Supplementary File**

**Variables:** Health records will be linked to extract the following information on mothers with a son or daughter with intellectual disabilities (and on the matched general population).

| **Table 1.** Variables of interest **Data Sources** | | **Variables and codes** | **Timescale** |
| --- | --- | --- | --- |
| Census 2011 | | Scottish Index of Multiple Deprivation (based on post-code): SIMD rank | 27.3.11 |
| Census 2011 | Scottish Index of rurality (three items): Urbanrural8Fold2011-12Code | | |
| Census2011 | | Who usually lives in house: HHCHUK11 and AHCHUK11 | 27.3.11 |
| Census2011 | | Household size: AHCHUK11 | 27.3.11 |
| Census2011 | | Accommodation type: TYPACCOM | 27.3.11 |
| Census2011 | | Tenure of household: TENHUK11 | 27.3.11 |
| Census2011 | | Landlord type: LANDLORD | 27.3.11 |
| Census2011 | | Number of cars at household: CARSNO | 27.3.11 |
| Census2011 | | Relationship of household members: RELHRPPUK11 | 27.3.11 |
| Census2011* | | Gender: SEX | 27.3.11 |
| Census2011* | | Date of birth (month and year only): DOB | 27.3.11 |
| Census2011 | | Marital and civil partnership status: MARSTAT | 27.3.11 |
| Census2011* | | Are you in full-time education (for children in household):STUDENT | 27.3.11 |
| Census2011* | | Country of birth: COB | 27.3.11 |
| Census2011 | | Provision of unpaid care: CARERPS11 | 27.3.11 |
| Census2011 | | Religion:RELPS11 | 27.3.11 |
| Census2011* | | General health: HEALTH | 27.3.11 |
| Census2011* | | Conditions: mental health, autism, deaf, blind: NATCON | 27.3.11 |
| Census2011* | | Long-term health problem or disability: DISABILITY | 27.3.11 |
| Census2011 | | Qualifications: HLQPS11 | 27.3.11 |
| Census2011* | | Employment status: EMPSTAT | 27.3.11 |
| Census2011* | | Economic activity: ECOPUK11 | 27.3.11 |
| Census2011* | | Activity last week: ACTLW | 27.3.11 |
| Census2011* | | Have you ever worked: EVERWORK | 27.3.11 |
| Census2011* | | How many hours a week do you work: HRSWRKD and Hours worked (grouped) HRSWRKD_GROUP | 27.3.11 |
| PIS* | | BNF Codes: Hypnotics: (4.1), Anxiolytics (4.1), Antidepressants (4.3) | 27.3.10-28.3.17 |
| SMR04 | | Admission Date (6 digits) | 27.3.10-28.3.17 |
| SMR04 | | Discharge Date (6 digits) | 27.3.10-28.3.17 |
| SMR04 | | Mental health diagnosis ICD-10 codes: F30-F39 Mood affective disorders; F40-F48.9 Neurotic, stress-related and somatoform disorders; F50-F50.9. Eating Disorders; F51-F51.9 Sleep | 27.3.10-28.3.17 |
| SMR04 | | Inpatient episode | 27.3.10-28.3.17 |
| SMR04 | | Any previous psychiatric care: Code 1, 2, 3, 9. | 27.3.10-28.3.17 |
| NRS | | Death of individual with intellectual disabilities | 28.3.11-29.3.17 |

Variables with * will be extracted for both mothers and the son or daughter with intellectual disabilities.
